# Exosomes Modulate the Viral Replication and Host Immune Responses in HBV Infection

**DOI:** 10.1155/2019/2103943

**Published:** 2019-05-28

**Authors:** Shuang Li, Shilin Li, Shaobo Wu, Limin Chen

**Affiliations:** ^1^Provincial Key Laboratory for Transfusion-Transmitted Infectious Diseases, Institute of Blood Transfusion, Chinese Academy of Medical Sciences and Peking Union Medical College, Chengdu, Sichuan 610052, China; ^2^Toronto General Research Institute, University of Toronto, ON, Canada M5G 1L6

## Abstract

Although current diagnosis and treatment of hepatitis B virus (HBV) infection can maintain viral suppression, new therapies need to be invented to sustain off-treatment virologic suppression and reduce side effects. Exosomes act as intercellular communicators to facilitate direct transfer of proteins, lipids, and nucleic acids between cells* in vitro* and* in vivo*. Pioneering work has demonstrated that exosomal cargos changed markedly during HBV infection. An improved understanding of the functions of exosomes during HBV infection could lead to a powerful new strategy for preventing and treating HBV. In this review, we point out the role of exosomes in HBV infection: (1) exosomes could directly participate in HBV replication; (2) exosomes modulate immune response during HBV infections; (3) exosomal RNAs and proteins might be selected as novel biomarkers for the diagnosis of HBV infections; and (4) exosomes can also be designed as vaccines.

## 1. Introduction

Since Blumberg et al. discovered the hepatitis B virus (HBV) in 1965 [1], much has been elucidated regarding its genome, sequence, epidemiology, and hepatocarcinogenesis. HBV belongs to hepadnaviridae and mainly infects hepatocytes [2]. Despite much has been yielded in the effort to discover effective treatments for HBV, the interferon *α* (IFN-*α*) and Nucleotide Analogs (Lamivudine, Telbivudine, Entecavir, Adefovir, and Tenofovir) have been proved as the effective treatment for HBV chronic infection [3, 4], HBV infection continues to be a significant public health problem worldwide. Approximately, more than 2 billion people were infected by HBV and the infection appeared more frequently in the Asia-Pacific region. Though the exact pathogenesis of HBV is not yet completely understood, the current thought is that the virus replication is not directly cytotoxic to hepatocytes, but rather, it is the immune response that mediates the damage of infected liver cells [5, 6].

**Figure 1:**
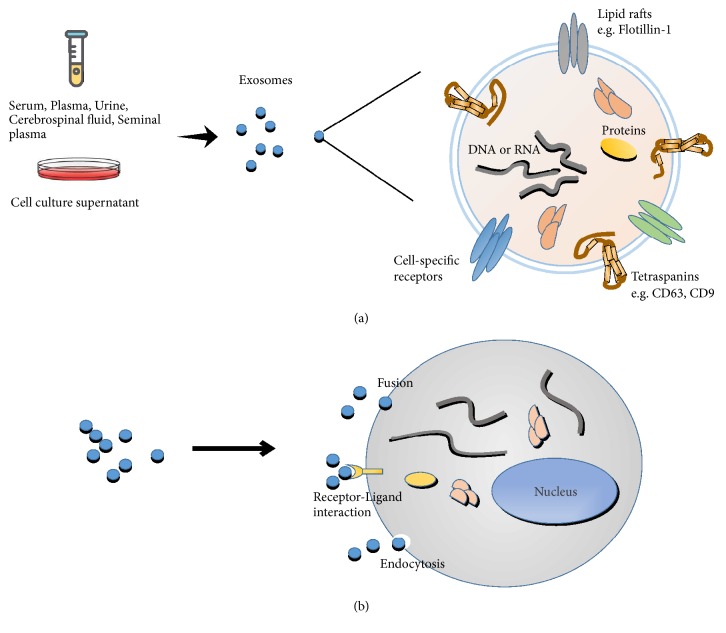
The characteristics of exosomes. (a) Exosomes are nanovesicles originated from membranes, the diameter from 30 nm to150 nm. In normal and pathological conditions, cells release exosomes to extracellular matrix and can be detected in many types of body fluids (serum, plasma, lymph, cerebrospinal fluid, saliva, urine, tears, sweat, etc.) and cell culture supernatants. (b) Exosomes play the role of information transactors among cells through three ways: (1) fusion directly with the target cell membrane; (2) the exosomal ligands binding to receptors of target cell; (3) the soluble components of exosomal proteins active endocytosis of target cells. Then, exosomes transfer bioactive nucleic acids, proteins, and lipids to target cells.

Exosomes are multiform, 30-150 nm diameter cup-shaped vesicles which are secreted by almost all types of cells. Exosomes can be found in most body fluids (like serum, plasma, lymph, saliva, urine, tears, sweat, semen, cerebrospinal fluid, and breast milk), as well as cell culture supernatants [7]. Exosomes have variable components which can reflect the status of host cells, and many researches found that exosomes can encapsulate lipids and multiple types of proteins including membrane fusion-related proteins, proteins involved in vesicle formation, integral membrane proteins, components of the major histocompatibility complex (MHC) classes I and II, proteins related to the cytoskeleton and cell metabolism, and cell surface proteins involved in oncogenesis [8, 9]. Additionally, nucleic acids including mRNA, miRNA, long non-coding RNAs (lncRNAs), and DNA have also been detected inside exosomes [10, 11]. In addition, the exosomal membrane helps to protect these cargos from enzymatic degradation, and exosomes have other attractive features, such as low immunogenicity, high biocompatibility, and the ability to overcome biological barriers [12]. So recent studies highlight the importance of exosomes in intercellular communication by transmitting biological signals between cells to regulate a diverse range of physiological and pathological processes. For example, neuronal exosome release is triggered by Ca^2+^ entry through* N*-methyl-D-aspartate (NMDA) and *α*-amino-3-hydroxy-5-methyl-4-isoxazolepropionic acid (AMPA) receptors at glutamatergic synapses, suggesting that exosome release may be part of normal synaptic physiology [13]. These exosomes can play the important role of cell communication on both neighboring and distal cells in the central nervous system (CNS). Besides, exosomes have been tightly linked to pathological processes to transport tumorigenesis factors such as CD97 which can activate of MAPK signaling pathway to promote gastric cancer (GC) cell proliferation and invasion [14], as well as the spread of autoantigens such as GAD65 and IA-2 which may accelerate diabetes onset* in vivo* by stimulating the autoimmune response [15]. Because exosomes with low immunogenicity, so* in vivo*, dendritic cells derived exosomes probably need a danger signal to be presented by an “exosome-presenting cell” for inducing an immunogenic response; without a danger signal, dendritic cells derived exosomes could mediate tolerance [12]. And studies found that during viral infections, exosomal cargos changed profoundly, and many researches indicated that exosomes isolated from virus-infected cells contain pathogen agents such as HCV RNA [16] and HIV-1 [17] which can, respectively, promote HCV and HIV infections [18, 19]. Meanwhile, some specific contents in exosomes can also play pivotal anti-infection roles through directly inhibiting viral replication and/or inducing antiviral immune responses [20]. Via exosomes transmitting and delivering, these functional components enter into recipient cells to process intracellular communication and various biological activities (Figure 1). In this review, we summarize the role and probable mechanisms of exosomes participating in cellular crosstalk during HBV infection.

## 2. Exosomes Participate in HBV Replication

Many enveloped viruses are reported to employ exosomes related proteins to form enveloped virions. HBV envelope proteins colocalize with multivesicular bodies (MVB, including exosomes) related proteins AIP1/ALIX and VPS4B, and either of these proteins being negative mutant can block the production and/or release of enveloped HBV virions [21]. Some viruses (like HCV and HIV) can hijack host exosomes; they can assimilate viral constituents into exosomes. And these exosomes distribute from infected cells transport various vital components to neighboring cells which help in regulating cellular responses and producing infections [22]. This has led to the proposal of the “Trojan exosome hypothesis,” and HBV and exosomes can have similarities in their biogenesis (Table 1). HBV RNA and viral DNA were detected in CD81^+^ exosomes from HepG2 cells with pHBV [23]. And exosomes derived from CHB patients serum also contained HBV DNA (rcDNA and cccDNA) and RNA (HBx and HBs/p), as well as HBsAg. And these exosomes can transmit HBV to uninfected hepatoma cells. Furthermore, exosomes derived from CHB patients suppress the cytotoxicity of NK cells, the expression of degranulation molecule CD107a and activating receptor NKp44, and the production of IFN-*γ* and the tumor necrosis factor (TNF)-*α*. These data suggest that exosomes derived from CHB patients can transmit HBV infection as free virus and depress the function of NK-cell dysfunction [24]. Several proteins encoded by the HBV genome, including large S, core and P proteins, were found inside exosomes isolated from an HBV-inducible cell line HepAD38. Besides, the differences of exosomal protein contents secreted by the HepAD38 cell line with or without HBV replication were evaluated by label-free proteomic analysis; 1412 protein groups were identified and bioinformatic analysis revealed that 32.98% exosomal proteins were plasma membrane-associated proteins as well as some proteins participating in the regulation of cytokine production or mediating signaling pathways. These data may provide insights into the potential function of exosomes in HBV-host interaction and immunomodulatory effect during HBV infection [25]. Since the first report of EBV can encode miRNAs (such as ebv-miR-BHRF1-1 and ebv-miR-BART1), it has been demonstrated that many viruses, like HIV and HCV, can encode specific viral miRNAs which play important roles in diverse cellular processes, including interactions between virus and host [26]. An HBV-encoded miRNA (called HBV-miR-3) was identified by deep sequencing, the HBV-miR-3 expression was detected in patients with HBV infection, and the expression level was significantly higher in the sera of patients with HBV infection in the acute phase than in those in convalescence. Meanwhile, HBV-miR-3 was released into the circulation by exosomes isolated from HepG2.2.15 (a human liver hepatocellular carcinoma cell line which contains complete HBV genome and is capable of stable HBV expression and replication in culture). The exosomal HBV-miR-3 can target a unique site of HBV transcript to reduce the level of HBV pregenomic RNA (pgRNA) and HBV core protein (HBc) protein and finally inhibit the HBV replication and protein expression [27]. These data indicate that HBV-encoded miRNAs may control self-replication by targeting viral transcripts, and the process may contribute to HBV persistent infection in patients. HBV x (HBx) protein is required for viral infection and replication, and it is closely associated with the development of hepatocellular carcinoma (HCC). Moreover, both HBx mRNA and protein exist in exosomes isolated from the HBx-expressing hepatoma cells and mass spectrometry (MS) studies suggest that the entrapped HBx mRNA could be translated into viral protein in hepatic stellate cells. And HBx could increase the production of exosomes through inducing the activity of nSMnase2 (the key enzyme involved in exosomes biogenesis) [28]. These data presented establish that HBx can modulate the biogenesis of host exosomes and alter neighboring liver cells. During HBV infection, HBx selectively decreases intracellular APOBEC3G (A3G) protein level and its effects on intracellular A3G level by affecting its export through exosome production and secretion. A3G belongs to apolipoprotein B mRNA editing catalytic polypeptide-like (APOBEC) family which has been generally considered as a restricting factor of HBV infection [29, 30]. These data might provide a new insight into the mechanism of HBx-mediated activation of HBV which might involve regulating host restriction factor A3G. In addition, proteomic analysis data demonstrate the content of exosomal proteins change markedly isolated from Huh-7 cells (originated from human liver cells) infected with HBx, HBV, and HBV(HBx-), respectively, and these exosomal proteins display little overlap between each group. Furthermore, compared with HBV negative controls, exosomal proteins isolated from HBV-infected patients showed specific changes. These exosomes contained higher levels of HCC related proteins, like heat shock protein 90B1 (HSP90B1) and valosin containing protein (VCP) [31]. Even these results highly illustrated that exosomes contribute to HBV spread and can modulate host biological activities, but whether exported components contained in exosomes have any effects on anti-HBV defense of recipient cells still needs future investigations, and the interaction between HBV induced exosomes and host is also worth pursuing.

## 3. Exosomes Modulate Immune Response during HBV Infection

**Figure 2:**
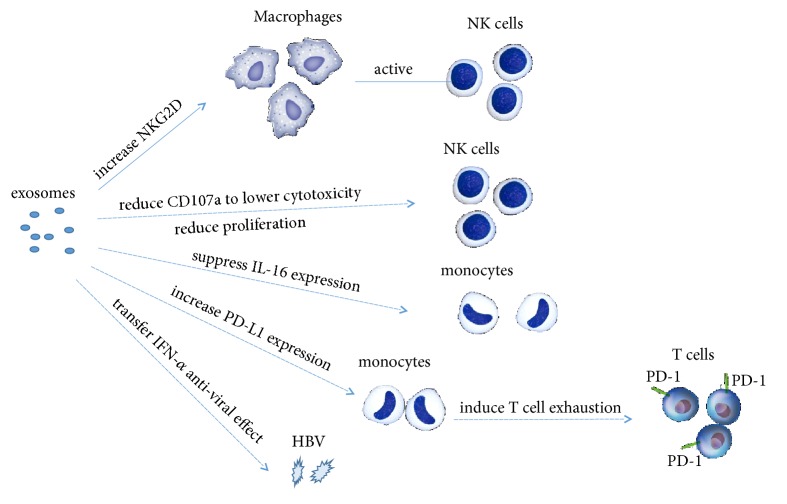
Exosomes modulate immune response during HBV infection. On the one hand, exosomes can play anti-HBV infection roles by increasing macrophages and NK cells function and delivering antiviral molecules among cells. On the other hand, HBV induced exosomes can promote HBV infections by inhibiting immune responses directly or indirectly and influencing cytokine-mediated signaling pathways and cytokine production.

Exosomes can elicit immune response as well as serve to transfer pathogens to their reservoirs in order to support latency. Therefore, altered exosomal components could determine the fate of viral infection and disease progression [32]. CD81^+^ exosomes released from hepatocytes with pHBV induced NKG2D ligand expression in macrophages through MyD88, TICAM-1, and MAVS-dependent pathways; these data suggest the importance of exosomes for macrophage function. Moreover, exosomal miR-21 and miR-29a, as well as other immunosuppressive miRNAs levels, were markedly increased by HBV infected HepG2-NTCP cells. miR-21 downregulates IL-12p35 mRNA expression and miR-29a is known to suppress IL-12p40 mRNA expression. So these exosomal miRNAs may inhibit NK cells activity through downregulating IL-12 expression [23]. These observations indicate that exosomes play a crucial role in the innate immune response against HBV. And exosome would mediate viral escape from the host innate immune response through downregulating IL-12. When NK cells from health donors co-cultured with exosomes derived from chronic HBV infection patients sera, the HBV rcDNA and HBV RNA can be transmitted into NK cells. And these transmitted HBV components can reduce CD107a (a cytotoxicity mediator of NK cells) to lower cytotoxicity of NK cells, as well as the proliferation of NK cells [24]. This means HBV-induced exosomes can influence the function and survival of NK cells. Therefore, the intricate relationships among various viral components and host factors could determine whether viral clearance or persistence occurs. Besides, exosomes can deliver proteasome subunit proteins to monocytes. HepAD38, the HBV-inducible cell line, secreted exosomes contain higher proteasomal activity proteins. Transmitting via exosomes these specific proteins can suppress IL-6 expression in monocytes [25]. These data demonstrated that HBV-induced exosomes might influence the production of proinflammatory molecules in the recipient monocytes. In addition, exosomes isolated from HepAD38 cells with HBV replication can strongly upregulate programmed-death ligand 1 (PD-L1) expression in monocytes compared with exosomes secreted from HepAD38 cells without HBV replication [33]. Programmed-death protein 1 (PD-1) expressed on T cells binding with PD-L1 induces the T cell exhaustion and protects target tissues from immune-mediated damage. So HBV-induced exosomes might promote HBV infection by suppression of T cells. Type I interferons (IFNs), mainly interferon-*α* (IFN-*α*) and IFN-*β*, serve as an important role in controlling viral replication during the initial stages of infection [34]. IFN-*α* can induce the transfer of resistance to HBV from nonpermissive liver nonparenchymal cells (LNPCs) to permissive hepatocytes via exosomes. Some specific antiviral activity molecules can be sorted into exosomes from IFN-*α*-treated LNPC. These antiviral molecules can be transferred through internalizing exosomes to hepatocytes and can attenuate HBV replication in hepatocytes [35]. Also, viral antigen expression and DNA quantification results indicate that exosomes isolated from IFN-*α* treated macrophages can efficiently transfer IFN-*α*-induced anti-HBV activity to HepG2.2.15 cells [36, 37]. IFNs function as natural antiviral mechanisms and have various therapeutic applications. After binding to the interferon receptor complex (IFNAR1-IFNAR2), IFN-*α* and IFN-*β* can be signalled through a kinase of the Jak family to the signal transduction and activator of transcription (STAT) pathway to the transcription of interferon-stimulated genes (ISGs: ISG15, ISG56, MxA, etc.), which are under the control of interferon-stimulated response elements (ISREs) [38]. The expression of ISGs establishes an antiviral state in host cells during viral infections. A previous study demonstrated that human liver endothelial cells could release ISG-enriched exosomes, which can inhibit HCV replication in liver parenchymal hepatocytes [39]. Later, exosomes from human brain microvascular endothelial cells were identified which contain antiviral factors including several key ISGs, such as ISG15, ISG56, and Mx-2 [40]. These data and findings have introduced and expanded the idea that exosomes enriched with ISGs may be vital in sensing and controlling viral infection. Whether HBV infection can induce ISGs enrichment in exosomes remains to be determined. As mentioned, exosomes and HBV are mutually influenced and mutually stimulated, and the function of exosomes during HBV infection is like a double-edged sword (Figure 2).

## 4. Exosomes as Markers for HBV Diagnosis

As described above, exosomes are involved in multiple steps during HBV infection; quantitative and qualitative analyses of the differences in the composition of exosomes in health and infected patients have been extensively reported. These differences, together with easy isolation and relatively stable for storage, make exosomes become excellent biomarker reservoirs as well as potential applications for diagnosis [41]. Recent studies identified some specific exosomal cargos as biomarkers for different liver diseases [42]. The level of HBV DNA and serum alanine aminotransferase (ALT) has been commonly used in estimating liver disease and as the important criterion for defining which patients need therapy. However, relying on serum ALT levels as a prerequisite to choosing treatment candidacy has limitations [43]. For example, 18 exosomal miRNAs (like hsa-miR-221-3p, hsa-miR-25-3p) were upregulated and 6 exosomal miRNAs (like hsa-miR-372-3p, hsa-miR-10a-5p) were downregulated in persistently normal ALT (PNALT) patients with the liver tissue inflammation. So these exosomal miRNAs are more sensitive than ALT to assess liver inflammation in the CHB patients with PNALT [21]. In addition, the levels of hnRNPH1 mRNA and miR-21 in serum exosomes isolated from HCC patients were remarkably higher than in CHB group. Receiver operating characteristic (ROC) curve analyses showed that exosomal hnRNPH1 mRNA level can discriminate HCC from CHB. The detection of serum exosomal miR-21 is also more sensitive than in serum, so hnRNPH1 mRNA and miR-21 in exosomes may serve as sensitive and specific biomarkers to diagnose HCC and distinguish CHB [44, 45]. The interest in using miRNAs within circulating exosomes as noninvasive biomarkers has increased rapidly; because of the exosomal lipid bilayer membrane, miRNAs are protected from degradation and keep stable in the body fluids. Despite the great benefits of exosomal miRNAs in diagnosis, there are several issues that still need to be addressed. Firstly, the selection of suitable reference genes as normalization factors is necessary to accurately compare exosomal miRNA transcripts. In particular, U6 (CCG-1) or miR-181a (RG-5d) had lower sensitivity for the comparability of miR-21 expression between CHB patients and HCC patients. The combination of miR-221, let-7a, miR-191, miR-26a, and miR-181a (RG-5d) was the optimal reference gene set, for the comprehensive investigation into the progression of CHB to HCC [46]. These findings highlight the importance of validating reference genes before quantifying target miRNAs. Secondly, biological body fluids are the rich source of exosomes with different origins, which make it difficult to isolate HBV-induced exosomes. The specificity of exosomal miRNAs still needs to be validated. For example, exosomal miR-21 is also highly expressed in glioblastomas pancreatic cancer, colorectal cancer, breast cancer, and colon cancer [47–49]. Therefore, large-scale studies of HBV and HCC patients would determine the value of differentially expressed exosomal miRNAs as potential biomarkers for distinguishing HBV patients from HCC. CHB patients on long-term treatment with nucleoside or nucleotide analogs are at risk of selecting drug-resistant HBV mutation strains. In two cases of HBV-infected patients whose treatment with entecavir (ETV) and tenofovir (TDF) is ineffective, rtS78T mutation was found in the reverse transcriptase (RT) gene of the HBV genome. The mutation causes a premature stop codon at sC69 and thereby deletes almost the entire small HBV surface protein in viral particles and exosomes. These changes facilitate replication and resistance to ETV and TDF treatment [50]. Further studies are needed to predict drug resistance according to specific changes in exosomal contents. With the deep study of these correlations between exosomal components and diseases, exosomes gradually become potential diagnostic and prognostic tools.

## 5. Exosomes as New Therapies for HBV Infection

As the carriers of functional RNAs and proteins, exosomes have attracted much attention as novel targets to develop new drugs. As potential vehicles, exosomes can also deliver some therapeutic agents, and the therapeutical molecules can be loaded into exosomes using either passive or active means. It was found that exosomes isolated from lipopolysaccharide endotoxin (LPS)-stimulated human monocytic cell line (THP-1) can induce a proinflammatory profile in healthy mice through the induction of cytokines such as tumor necrosis factor alpha (TNF-*α*), chemokine (C-C motif) ligand 5 (CCL5), and interleukin 1 beta (IL-1*β*). Moreover, when these exosomes are used as adjuvants for hepatitis B recombinant antigen (HBsAg), the cellular immune response is induced in mice and triggered an immunomodulatory effect on the cellular immune response by increased IFN-*γ* concentration and hastened the appearance of IgG antibody production [51]. These results showed that the unmodified exosomes can trigger immunostimulatory effect which could make them attractive coadjuvants. Engineered exosomes can be loaded with specific molecular and delivery* in vivo* for treating diseases. Based on the fusion between exosome-anchoring protein Nef mutant (Nefmut) and HBV core protein, elicited exosomes contain huge amounts of HBV core protein. When translated in animals, cytotoxic T lymphocyte (CTL) immunization against HBV can be induced by the engineered exosomes. The activated HBV-specific CTLs reconstitution can be of significant therapeutic effect, so these engineered exosomes open a new way for vaccine candidates against HBV [52]. As further investigations on exosomes, identification and delivery of specific antiviral molecules or therapeutic agents through exosomes will be the potential therapeutic strategy for HBV treatment and control, and many additional tests will be necessary to apply them in therapy.

## 6. Conclusion

**Figure 3:**
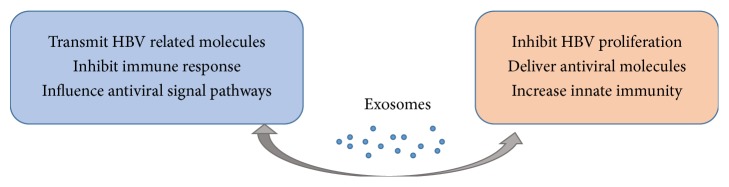
The dual effects of exosomes in HBV transmission and antiviral response. HBV induced exosomes containing viral proteins and RNAs can promote infection in three ways: (1) causing further infection by transmitting viral-related molecules; (2) inhibiting immune responses directly or indirectly; (3) influencing cytokine production and cytokine-mediated signaling pathways, while exosomes can play anti-infective roles by (1) inhibiting pathogen proliferation and infection directly; (2) delivering antiviral molecules among cells; and (3) increasing monocyte-macrophages and NK cells function. There must be a balance between infection and anti-infection processes, and exosomes as crucial messengers might modulate this balance in different ways as discussed in the present review.

Exosomal vesicles can transmit signals between pathogens and the host cells regarding various aspects of the host defense. In this review, we focus on the exosome functions in the relationship with HBV infection and anti-infection. On the one hand, the exosomes secreted by HBV infected cells are responsible for the transmission of viral information, and the exosomal cargos may be involved in viral transmission and pathogenicity. On the other hand, exosomes can transport antiviral agents and induce antiviral response (Figure 3). Through modulating immune response, exosomes may play promoting or limiting role in the process of HBV infection, so they can be used to develop preventive and therapeutic vaccines. In addition, because exosomes originated from all cell types, the levels of nucleic acid and proteins encapsulated in exosomes will change under pathological conditions, so exosomes in body fluids can be used as noninvasive markers which have potential application prospects in early diagnosis of HCC from CHB, prediction of drug resistance, and so on.

As the understanding of exosomes functions in the process of HBV infection is still in the early phase, identification of exosomal proviral and antiviral components for cell-cell communications during HBV infection needs to be further clarified. Further functional analyses of exosomal cargoes may be important in understanding the mechanisms of HBV infection and identifying sensitive therapeutic targets.

## Figures and Tables

**Table 1:** HBV elements packaged within exosomes and their effects.

HBV element		Source	Effect	Ref.
HBV DNA	rcDNA	CHB patients serum	Transmit HBV infection	[23, 24]
	cccDNA			

HBV RNA	HBx	HBx-expressing hepatoma cells; CHB patients serum	Transmit HBV infection	[23, 24, 28]
	HBs/p			

HBV surface protein	HBsAg	CHB patients serum	Transmit HBV infection	[24]

HBV-encoded proteins	Large S	HepAD38 cell line with HBV replication	Transmit HBV infection	[25]
	Core protein			
	P protein			
	HBx protein	HBx-expressing hepatoma cells	Support viral spread and pathogenesis; export of intracellular A3G via exosome	[28, 29]

HBV-encoded miRNA	HBV-miR-3	HBV infected patients sera; HepG2.2.15 cells	Regulate HBV replication	[27]
